# A 48 SNP set for grapevine cultivar identification

**DOI:** 10.1186/1471-2229-11-153

**Published:** 2011-11-08

**Authors:** José A Cabezas, Javier Ibáñez, Diego Lijavetzky, Dolores Vélez, Gema Bravo, Virginia Rodríguez, Iván Carreño, Angelica M Jermakow, Juan Carreño, Leonor Ruiz-García, Mark R Thomas, José M Martinez-Zapater

**Affiliations:** 1 Departamento de Genética Molecular de Plantas, Centro Nacional de Biotecnología, CSIC, C/Darwin 3, 28049 Madrid, Spain; 2 Instituto de Ciencias de la Vid y del Vino (CSIC-Universidad de La Rioja-Gobierno de La Rioja). Complejo Científico Tecnológico. C/Madre de Dios 51. 26006 Logroño. Spain; 3 Instituto Madrileño de Investigación y Desarrollo Rural, Agrario y Alimentario (IMIDRA). Finca "El Encín". Ctra A2, Km 38.200. 28800 Alcalá de Henares. Madrid. Spain; 4 Instituto Murciano de Investigación y Desarrollo Agrario y Alimentario (IMIDA). Estación Sericícola. C/Mayor, s/n. 30150 La Alberca. Murcia. Spain; 5 CSIRO Plant Industry, PO Box 350, Glen Osmond, SA 5064, Australia; 6 Instituto Nacional de Investigación y Tecnología Agraria y Alimentaria. Ctra de A Coruña, Km 7. 28040. Madrid. Spain; 7 Instituto de Biología Agrícola de Mendoza, Facultad de Ciencias Agrarias, CONYCET-Universidad Nacional de Cuyo, Almirante Brown 500, M5528AHB Chacras de Coria, Argentina

## Abstract

**Background:**

Rapid and consistent genotyping is an important requirement for cultivar identification in many crop species. Among them grapevine cultivars have been the subject of multiple studies given the large number of synonyms and homonyms generated during many centuries of vegetative multiplication and exchange. Simple sequence repeat (SSR) markers have been preferred until now because of their high level of polymorphism, their codominant nature and their high profile repeatability. However, the rapid application of partial or complete genome sequencing approaches is identifying thousands of single nucleotide polymorphisms (SNP) that can be very useful for such purposes. Although SNP markers are bi-allelic, and therefore not as polymorphic as microsatellites, the high number of loci that can be multiplexed and the possibilities of automation as well as their highly repeatable results under any analytical procedure make them the future markers of choice for any type of genetic identification.

**Results:**

We analyzed over 300 SNP in the genome of grapevine using a re-sequencing strategy in a selection of 11 genotypes. Among the identified polymorphisms, we selected 48 SNP spread across all grapevine chromosomes with allele frequencies balanced enough as to provide sufficient information content for genetic identification in grapevine allowing for good genotyping success rate. Marker stability was tested in repeated analyses of a selected group of cultivars obtained worldwide to demonstrate their usefulness in genetic identification.

**Conclusions:**

We have selected a set of 48 stable SNP markers with a high discrimination power and a uniform genome distribution (2-3 markers/chromosome), which is proposed as a standard set for grapevine (*Vitis vinifera *L.) genotyping. Any previous problems derived from microsatellite allele confusion between labs or the need to run reference cultivars to identify allele sizes disappear using this type of marker. Furthermore, because SNP markers are bi-allelic, allele identification and genotype naming are extremely simple and genotypes obtained with different equipments and by different laboratories are always fully comparable.

## Background

Grapevine (*Vitis vinifera *L.) is one of the most valuable horticultural crops in the world. Many of the widely cultivated varieties are very ancient genotypes that have been vegetatively multiplied for centuries and spread worldwide. In many places the same genotypes were re-named leading to synonyms (different names for the same variety) as well as homonyms (different varieties identified under the same name). Currently, there is a large but imprecise number of grapevine varieties in the world (several thousands, [1]): This number could likely be reduced once all varieties are properly genotyped and compared.

When genetic identification is taken into account, two goals have to be fulfilled: i) the availability of a large enough number of polymorphic markers; and ii) the existence of public genotype databases allowing for comparisons with previously characterized genotypes. Markers should provide a high discrimination power and yield reproducible genotype data among different laboratories and detection platforms as well as over time. Markers should also be stable, meaning that they produce consistent and repeatable results after repeated propagation of the varieties. This is especially important in the case of grapevine where many varieties have been under cultivation for centuries, and some molecular markers have been shown not to be fully stable in certain old varieties, due to somatic mutation [2]. In addition, genotyping methodologies should be easily accessible at low cost and comparable and genotype data should be easily stored in databases and publicly accessed.

Grapevine genotyping is currently based on microsatellite markers or simple sequence repeats (SSR), which have been very useful not only for genetic identification [3] but also for parentage analysis [4]. These markers have some relevant advantages for research such as their co-dominance, multi-allelism and high levels of polymorphism [5]. However, there are a number of disadvantages in using SSR markers. The most important problem is related to allele binning: The process that converts raw allele lengths into allele classes normally expressed by integer numbers [6]. Problems stemming from allele miscalling derive in part from the wide use of SSR based on di-nucleotide repeats and the frequent addition of one Adenine nucleotide by the DNA polymerase, which gives rise to alleles very close in size and difficult to distinguish. This problem can be partially solved with the use of SSR with core repeats three to five nucleotides long such as those recently developed, based on the information provided by the whole genome sequence [7]. However, even if longer repeat length markers are used, it is also important to take into account the fact that different analytical systems (e.g. DNA sequencers of different brands) could produce different allele sizes and consequently different bins, increasing the hardship of comparing genotype tables produced by different laboratories. To overcome these difficulties, standardization and exchange of information concerning grapevine genetic resources using reference varieties for certain microsatellite markers and alleles have been proposed [6] and discussed within European Projects such as GENRES 081 and Grapegen06, aiming at integrating genotypic information obtained by different laboratories.

In recent years, numerous sequencing projects have generated an abundance of sequence information and nucleotide polymorphisms. These belong to two basic types: single nucleotide polymorphisms (SNP) and insertions-deletions of different lengths (INDEL). Among them, SNP markers have the advantage that they are mostly bi-allelic and are very frequent in genomes. Although SNP polymorphism information content (PIC) is lower than that of SSR markers, tens, hundreds or even thousands SNP can be easily used when required. SNP are highly reproducible among laboratories and detection techniques, since the different alleles are not distinguished on the basis of their size but on the basis of the nucleotide present at a given position. All these features and their unlimited availability are making SNP the markers of choice for the development of identification panels in many animal and plant species [8-12].

In this work, we characterized the genetic features of 332 SNP to select a panel of 48 markers suitable for cultivar identification in grapevine. We show here that the panel has a similar discrimination power as a set of 15 SSR markers and can represent a very robust genetic identification system, problem-free of allele miscalling among laboratories or detection technologies. We also demonstrate that markers have a very low genotyping error rate, a low rate of appearance of new mutations when compared to SSR, and are amenable for easy storage in genotype databases. Given the state of revision and integration of genetic resources in grapevine, our SNP panel may become a rapid tool for genetic identification and genotype calling in the crop.

## Results and Discussion

### Single Nucleotide Polymorphisms (SNP) Detection

Identification of SNP markers in the grapevine genome was carried out based on a re-sequencing strategy in a selected sample of grapevine genotypes as previously described [13]. The sample was chosen to include non-related wine and table grape cultivars of ancient origin as well as wild accessions. Based on the available information, cultivars corresponded to different genetic groups [14] and had chlorotypes belonging to the four major types described in grapevine [15]. A total of 270 SNP markers were identified in this way to which we added 62 SNP validated at CSIRO across a range of genotypes. For the final 332 SNP we developed genotyping strategies based on SNPlex™. A first step to analyze the quality of these polymorphisms in grapevine and to estimate their allele frequencies was to genotype a sample of 300 accessions of grapevine including wine and table grape varieties as well as wild accessions (Additional file 1, Table S1). This approach allowed for discarding 61 SNP that did not worked in the analyses and 33 that, although initially identified as polymorphic in sequence comparisons, either behaved as monomorphic in the analyzed sample or were genotyped as heterozygous in 100% of the samples suggesting the existence of duplicated loci. As a result only 238 SNP markers were considered for further analyses (Additional file 1, Table S3).

### Genomic Location of SNP markers

Genotyping of four grapevine segregating progeny populations with the seven SNPlex™ sets allowed us to genetically map most of the 238 polymorphic SNP, which were heterozygous in one or both parents in at least one of the progeny populations (Additional file 1, Tables S4 and S5). On average, the use of the seven SNPlex™ sets allowed for including 114 markers in the consensus map of any given mapping population: 42 for each progenitor (segregation types aaxab and abxaa) and 29 common markers (abxab).

**Figure 1:**
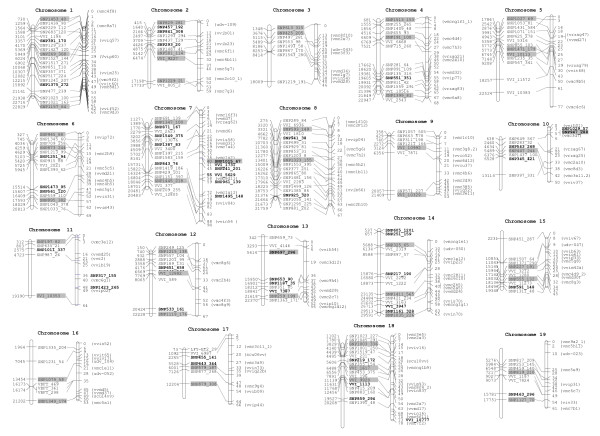
**SNP genetic and physical position**. For each chromosome, the map on the left (gray bars) shows the physical position of studied SNP markers on the 12X grapevine sequence of the PN40024 near homozygous line [40] indicated in kilobases; and the map on the right (empty bars) shows the genetic position, indicated in centiMorgans, of microsatellites (between brackets) and SNP genetically mapped using the four segregating progenies. Markers with known position in only one of these maps are indicated in bold: in the map on the left, the SNP with known physical position that could not be mapped genetically; and in the map on the right SNP mapped genetically but with unknown or uncertain physical position.

The integrated map developed for the eight parental cultivars included 168 microsatellites and 202 SNP (85% of the polymorphic SNP) allowing for identifying the relative positions of markers not segregating in the same progeny population (Figure 1, Additional file 1, Table S3). Three additional segregating SNP could not be mapped due to inconsistencies in linkage analyses (Additional file 1, Table S3). Molecular markers were distributed along all 19 chromosomes with an average distance between adjacent markers of 3.4 cM (5.7 when considering only SNP). The integrated map had a total size of 1204 cM (Additional file 1, Table S4), similar to other complete linkage maps published for *Vitis vinifera *[16-19]. Because the integrated map was based on mean recombination frequencies [20] and a total of 313 progeny individuals was considered, it should provide a good estimation of genetic distances. However, the accuracy of the genetic position assigned to each marker is limited by the number of progenies in which it is segregating, the segregation types in each progeny, the presence of markers with distorted segregations and the possible existence of differences in recombination rates among the progenitor cultivars. Sixty-seven percent of the 202 SNP markers mapped were segregating in more than one mapping population (25%, 27% and 15% in two, three and four, respectively) and only 11 SNP showed the less informative segregation type < abxab >. Finally, distorted segregation rates were low in Dominga × Autumn Seedless, Monastrell × Cabernet Sauvignon and Muscat Hamburg × Sugraone crosses (ranging between 7 and 12%), but higher in Ruby × Moscatuel (23%), which is likely due to the smaller size of the progeny (Additional file 1, Table S4).

Sequence searches for the SNP surrounding sequences (Additional file 1, Table S3) within the 12× genomic sequence of *Vitis vinifera *http://www.genoscope.cns.fr/externe/GenomeBrowser/Vitis/ allowed for physically positioning most of the studied SNP (Figure 1, Additional file 1, Table S3). Two-hundred and twenty-five out of the 238 polymorphic SNP could be positioned on the physical map with an average of 12 SNP per chromosome (from 7 SNP on linkage groups 10, 11, 16 and 17, to 21 SNP on linkage group 8). The average distance among physically mapped SNP was 1.76 Mb. Thirteen SNP could not be physically located. This could be either due to the lack of significant matches with the 12× genomic sequence (VV5629 and SNP575_128), the identification of different locations with the same likelihood (SNP241_201 and SNP1495_148) or their localization on unlinked chromosome scaffolds. Linkage mapping allowed for localizing 12 out of the 13 SNP that could not be positioned in the physical map (Additional file 1, Table S3, Figure 1). The only marker that could not be mapped either physical or genetically (SNP575_128) corresponds with one of the two SNP where adjacent sequences could not be found in the search on the 12× genome sequence.

Marker order was generally conserved between physical and genetic maps, although discrepancies were found on chromosomes 1, 3, 10, 12 involving differences of up to 7.6 Mb and 12 cM. In addition, small local marker inversions, involving < 1.5 Mb and < 6 cM distances, were observed for chromosomes 1, 2, 4, 5, 6, 7, 8, 13 and 19 (Figure 1). Most of these discrepancies could be attributed to some of the previously mentioned factors affecting the accuracy of the genetic position assigned to each marker. However, none of these factors were present in the most important differences (chromosomes 3 and 10), which points out some problems in the current physical map of those regions and that may be related to genome rearrangements or assembly errors on the 12× grapevine sequence of the PN40024 near homozygous line http://www.genoscope.cns.fr/externe/GenomeBrowser/Vitis/. For example, marker SNP425_205 (one of the two SNP markers on chromosome 3 included in the SNP set for varietal identification) showed significant discrepancies between physical and genetic distances with the surrounding markers leading to differences in marker order for this region (Figure 1, Additional file 1, Table S3). In the current 12× version of the genomic sequence of *Vitis vinifera*, this marker is at 1.4 Mb from SNP613_315 (the second marker included in the 48 SNP set for varietal identification for this chromosome). However, marker order on the genetic map aligns with marker order in the version of the genomic sequence (8× at NCBI, data not shown) in which both SNP are separated by 4.4 Mb as well as with marker order in the Pinot Noir sequence http://genomics.research.iasma.it/gb2/gbrowse/grape/.

### Selection of the SNP Set for Genetic Identification

Currently, intra-laboratory genetic identification of grapevine varieties does not represent a major problem given the large number of microsatellite and SNP markers that have become available over the years [6,7,21-23]. However, it is very important to develop a system that is efficient, rapid and cheap for identifying the several thousand cultivars currently available in grapevine. This requires the careful design of a set of highly polymorphic and stable markers with proven quality and reproducibility that allow for constructing databases easy to share among different laboratories. In order to develop such a system based on SNP markers, three selection criteria were considered: high frequency of genotyping success, high minor allele frequency (MAF) to provide higher PIC and good chromosomal distribution to end up with a total of 48 SNP distributed at a rate of 2-3 SNP per chromosome. When these criteria were applied on the available SNP (Additional file 1, Table S3 and Figure 1), a selection that was used for the design of a 48 SNP set (Table 1) was obtained. A completely new design with only the selected 48 SNP set was built, and their stability and quality for genetic identification was thoroughly evaluated.

**Table 1:** Main features of the 48 SNP Set

		Physical position		Genetic position	
**SNP**	**Polymorphism**	**Chromosome**	**Nucleotide**	**LG**	**cM**

**SNP1003_336**	A/C	18	3829207	18	16.7
**SNP1015_67**	A/G	unknown	8839239	7	40.4
**SNP1027_69**	C/T	5	1785979	5	3.3
**SNP1035_226**	C/T	14	29590769	14	63.4
**SNP1079_58**	A/G	16	13454358	16	18.9
**SNP1119_176**	A/C	12	22228357	12	67.4
**SNP1127_70**	G/T	19	17751334	19	53.6
**SNP1157_64**	A/T	1	22828604	1	60.6
**SNP1215_138**	C/T	12	739916	12	21.9
**SNP1229_219**	G/C	2	17198115	2	52.7
**SNP1323_155**	A/C	8	13401437	8	36.6
**SNP1347_100**	A/G	7	1388822	7	0
**SNP1349_174**	A/G	16	21202286	16	50.1
**SNP1399_81**	A/G	4	21849155	4	66.5
**SNP1411_565**	A/T	14	23135445	14	38.1
**SNP1445_218**	A/G	7	18046355	7	81.7
**SNP1453_40**	A/G	1	729514	1	0.7
**SNP1471_179**	C/T	5	5773320	5	26
**SNP1513_153**	C/T	4	680574	4	0
**SNP191_100**	C/T	4	6409234	4	24
**SNP197_82**	A/C	11	311765	11	0
**SNP227_191**	A/C	15	15145042	15	21.3
**SNP259_199**	A/T	13	21618145	13	48.7
**SNP269_308**	A/G	1	5948674	1	29.3
**SNP325_65**	A/T	14	5687725	14	15.2
**SNP425_205**	A/C	3	3676120	3	29.9
**SNP447_244**	C/T	10	5489212	10	37.5
**SNP555_132**	A/C	15	18031506	15	34
**SNP579_187**	C/T	17	6000914	17	38.5
**SNP581_114**	A/G	2	5141894	2	24.6
**SNP593_149**	C/T	8	3320936	8	7.9
**SNP613_315**	C/T	3	1348328	3	0
**SNP697_296**	A/G	13	5613947	unknown	unknown
**SNP819_210**	A/T	19	7217380	19	42.1
**SNP829_281**	A/G	2	415342	2	0
**SNP873_244**	C/T	6	4258638	6	14
**SNP879_308**	A/G	17	12206201	17	64
**SNP895_382**	A/T	6	17593092	6	56.7
**SNP945_88**	A/G	6	327200	6	0
**SNP947_288**	A/G	unknown	9111477	10	4.8
**VV10113**	A/G	5	6744629	5	25
**VV10329**	C/T	9	21409416	9	53.1
**VV10353**	G/A	11	19390306	11	64.1
**VV10992**	A/T	9	3123999	9	14.1
**VV12882**	T/C	12	7768973	12	40.5
**VV1617**	A/C	18	6487636	18	27.9
**VV9227**	T/A	2	6474327	2	37.4
**VV9920**	A/G	18	11138668	18	48.5

### Evaluation of the Stability of the SNP Set for Genetic Identification

Stability of the 48 SNP markers was evaluated through the analysis of the genotypes obtained for an average of 85 plants for each 15 cultivars (Additional file 1, Table S2). This study also allowed for scoring the rate of genotyping success. The 15 cultivars represent a large phenotypic diversity for important traits in grapevine regarding their use (wine, table, and raisin), berry colour (black, red and white), maturity time (early, medium and late), presence of seeds (seeded and seedless) and other traits [24]. In addition to their diverse geographical origin (France, Spain, Near East, Middle East), the 15 cultivars exhibit age differences as well: from very ancient cultivars, likely more than thousand years old (e.g. 'Muscat of Alexandria', 'Thompson Seedless'), to cultivars originating only a few centuries ago (e.g. 'Cabernet Sauvignon' and those bred in the 20^th ^century (e.g. 'Cardinal', 'Crimson Seedless').

A total of 1342 plants were analyzed with the newly designed 48 SNP set. Table 2 shows the genotypes obtained for each variety. No genotype could be established in any of the plants for SNP VV1617 and, therefore, was excluded from the analysis. Nevertheless, this SNP worked regularly in other genotyping analyses and was included in further tests. In addition, genotyping for SNP325_65 and VV9227 failed completely in the 'Monastrell' cultivar. The genotype for SNP325_65 could be obtained for this cultivar after several analyses but this was not the case for VV9227 (data not shown). The existence of a homozygous null allele in this cultivar for VV9227 was discarded because it presented an A/T genotype for this SNP in the previous genotyping with the 332 SNP set.

**Table 2:** Genotypes for the 48 SNP set in the cultivars used for the stability study

	AIR	CBS	CAR	CRI	FLA	MER	MON	MOA	NAP	OHA	PAL	REG	SAU	TEM	THO
N° plants with complete genotype	70	56	79	80	55	77	75	86	82	84	81	64	64	58	54
SNP1003_336	AC	AA	AA	AC	AA	CC	AC	AC	AC	AC	AC	AC	AC	CC	AC
SNP1015_67	GG	GG	GG	AA	GG	GG	GG	GG	GG	AG	GG	GG	AG	GG	AG
SNP1027_69	CT	CC	CT	CT	CT	CC	CT	CC	CT	CT	TT	CC	CC	CC	CT
SNP1035_226	CT	TT	TT	CC	CT	TT	TT	CT	CC	CT	TT	CT	TT	CT	CT
SNP1079_58	AA	AG	AG	AA	AA	AA	AG	AG	AG	AA	AG	GG	GG	AG	AG
SNP1119_176	AA	CC*	CC	AA	CC	AA	CC*	CC*	AA	AA	CC*	CC*	CC	CC	CC
SNP1127_70	GG	GT	GG	GG	GG	GT	GG	GT	GG	GG	GG	GT	GT	GG	GG
SNP1157_64	TT	AT	AT	AT	TT	TT	TT	TT	AT	AT	TT	TT	AT	TT	TT
SNP1215_138	CC	CC	CT	CC	CC	TT	CT	CT	CT	CT	CT	CT	CT	CC	CT
SNP1229_219	CC	CC	CC	CC	CC	CC	CC	CC	CC	CC	CC	CC	CC	CC	CG
SNP1323_155	CC	CC	CC	AA	AA	AC	AA	AC	AA	AC	CC	CC	AC	AC	AC
SNP1347_100	AG	AG	AG	AG	AG	AG	AA	GG	GG	GG	AG	AG	AG	GG	AG
SNP1349_174	GG	AG	AA	AG	AA	AG	GG	AA	AG	AG	AG	GG	AA	AA	AG
SNP1399_81	AA	AG	AA	AA	AA	AA	AA	AA	AG	AA	AA	AA	AG	AA	AA
SNP1411_565	TT	TT	TT	TT	TT	AA	AT	AT	TT	TT	AT	AA	TT	AT	AT
SNP1445_218	AG	AA	GG	AG	GG	AA	AA	GG	GG	GG	GG	AG	AG	GG	AG
SNP1453_40	AA	AA	AG	AG	AA	AG	AA	AG	AG	AA	AG	AG	AG	GG	AA
SNP1471_179	TT	CT	CT	TT	TT	TT	TT	CT	CT	TT	TT	TT	CT	CT	TT
SNP1513_153	TT	CT	CT	TT	CT	CC	CT	TT	CT	CT	CT	TT	CC	CC	CT
SNP191_100	CC	CT	CC	CC	CC	CC	CC	CC	CC	CC	CC	CC	CT	CC	CC
SNP197_82	CC	AC	CC	AC	CC	AC	AA	CC	CC	CC	CC	CC	AC	AA	CC
SNP227_191	AA	AC	AC	AA	AA	AC	AC	AA	AA	AA	AA	AC	AA	AC	CC
SNP259_199	AT	AT	TT	AT	TT	TT	AT	AT	TT	AT	TT	AA	TT	AA	AA
SNP269_308	GG	GG	AG	AA	AG	AG	AG	AA	AA	AA	AG	AG	AG	GG	AA
SNP325_65	TT	AT	AT	AA	AA	AA	AT	AA	AA	AA	AT	AA	AA	TT	AA
SNP425_205	AA	AC	AA	AA	AA	CC	AA	AA	AA	AA	CC	AA	AC	AA	AA
SNP447_244	CT	CT	TT	CT	TT	CT	TT	CT	CT	CT	CC	CT	CT	CC	CT
SNP555_132	AA	AA	AC	AC	AA	AC	AC	CC	AA	AA	AA	AA	AC	AC	AA
SNP579_187	TT	TT	TT	CT	TT	TT	CT	TT	TT	TT	CT	TT	TT	TT	TT
SNP581_114	AG	AG	AA	AG	AG	AG	AG	AG	AG	GG	AG	AA	GG	AG	AG
SNP593_149	CT	CT	CT	CT	TT	CT	TT	CT	TT	TT	CT	TT	CT	TT	CT
SNP613_315	CT	CC	CC	CC	CC	CC	CT	CC	CC	CT	CT	CC	CC	CC	CT
SNP697_296	AG	AA	AA	AA	AA	AA	AA	AA	AA	AA	AA	AA	AA	AA	AA
SNP819_210	AT	TT	AT	TT	TT	AT	AA	AT	TT	AA	TT	TT	TT	AA	TT
SNP829_281	AG	AG	AG	AG	AG	AA	AA	AG	GG	AG	AG	AG	AG	AA	GG
SNP873_244	CT	TT	CC	CC	CC	CC	CT	CT	CT	CC	TT	CC	CT	TT	CT
SNP879_308	GG	AG	AG	AG	AA	AA	AG	AA	AA	AG	GG	AA	GG	AA	AA
SNP895_382	AT	TT	AT	AT	AT	TT	TT	AA	AA	AT	AT	AT	TT	AA	AT
SNP945_88	AA	AG	AA	AG	AA	AG	AA	AG	AG	AG	AG	AG	GG	AG	AG
SNP947_288	AG	AG	AG	GG	GG	AG	GG	AG	AG	AG	AG	GG	AG	AG	AG
VV10113	AA	AG	AA	AG	AA	AA	AA	AA	AA	AG	AG	AA	AA	AA	AG
VV10329	CT	CT	TT	TT	TT	CC	CT	CT	CC	CC	CT	CT	TT	CT	TT
VV10353	GG	AG	GG	GG	GG	AG	GG	AG	GG	GG	GG	AG	GG	GG	AG
VV10992	TT	AT	AT	AT	TT	AT	AT	AT	AT	AT	AT	TT	AA	TT	TT
VV12882	TT	CT	TT	TT	TT	TT	CC	TT	TT	TT	TT	TT	CC	TT	TT
VV1617															
VV9227	AT	AT	AT	TT	TT	TT	-	TT	TT	TT	AT	TT	AT	TT	TT
VV9920	GG	AG	AG	GG	GG	AG	GG	AA	AA	GG	GG	GG	GG	AA	GG

AIR: Airén; CBS: Cabernet Sauvignon; CAR: Cardinal; CRI: Crimson Seedless; FLA: Flame Seedless; ITA: Italia; MER: Merlot; MON: Monastrell; MOA: Muscat of Alexandria; NAP: Napoleon; OHA: Ohanes; PAL: Palomino; REG: Red Globe; SAU: Sauvignon Blanc; TEM: Tempranillo; THO: Thompson Seedless.

*The correct genotype is AC, according to data obtained later (see text)

A complete genotype (47 SNP) was obtained for 990 plants corresponding to an average of 66 plants per variety with a range from 54 to 86 plants (Table 2, Table 3) excluding 'Monastrell'. No genotype could be established for 65 plants. This could be due to a low DNA concentration in a number of cases (17 DNAs were below a concentration of 4 ng/ul) but, in most cases, failures were probably due to the presence of contaminants that prevented amplification. Apart from the cases where no plant (one SNP) nor SNP (65 plants) could be genotyped, the average genotyping rate was 97.1% (Table 3). Marker SNP697_296 presented the highest genotyping success rate and only failed in two plants. Ten SNP markers presented a genotyping success rate above 0.99, and 40 SNP above 0.95.

**Table 3:** Genotyping efficiency and reliability of the 48 SNP set

	N° Plants	Rate
Genotyped*	1277	
Complete genotype	990	0.775
> 95% genotype	1155	0.904
SNP highest genotyping success rate	1275 (SNP697_296)	0.998
SNP lowest genotyping success rate	1139 (SNP325_65)	0.892

	**N° individual SNP genotypes**	**Rate**

Total	60019	
Obtained	58256	0.971
N° mistaken genotypes	3	0.000051

* Excluding 1 SNP that did not work in this experiment and 65 plants for which none SNP genotype could be established.

Regarding the stability analysis, 99.4% of all the genotyped plants showed the genotype expected for the cultivar. Only three SNP showed a different genotype in plants of the same cultivar: SNP1119_176 and SNP581_114 (in one 'Ohanes' plant), and SNP1347_100 (in one 'Flame Seedless' plant). To determine if these variations were due to mutations (lack of stability) or genotyping errors, the analyses were repeated using the same DNA extraction as well as independent DNA extractions for each plant. The results indicate that all discrepancies corresponded to genotyping errors. In summary, no mutation could be found in the 58251 individual SNP genotypes established for the 15 varieties studied and, therefore, the SNP marker set could be considered highly stable.

### Evaluation of the SNP Set for Genetic Identification Purposes

A total of 200 grapevine accessions were genotyped with the 48 SNP set including a sample from each of the varieties studied in the stability analysis. Some of the accessions resulted in identical genotypes but these results always agreed with the expectations; since they corresponded either to synonymous cultivars or sports (phenotypically different cultivars generated by spontaneous somatic mutations and later propagated through cuttings). Sports are not expected to differ from their initial cultivar by using molecular markers. This was confirmed for several sports: 'Chasselas Apyrene', a seedless sport, did not differ from 'Chasselas Blanc'. Within the Pinot group, 'Pinot Blanc' showed an identical genotype for the 48 SNP set to 'Pinot Noir' and also 'Pinot Meunier', a genetic chimera [25], showed the same genotype. Nevertheless, 'Pinot Gris', another colour sport, presented a homozygous genotype CC for SNP1229_219, while the other cultivars of the group were heterozygous CG. This is not surprising since the 'Pinot' group has the largest intra-varietal variation measured with microsatellite markers [26-29].

Another one-allele difference was observed when genotypes obtained in this study were compared with those obtained for the same varieties in the stability analysis (see above) but, while in the case of 'Pinot Gris' the difference was consistent and could be considered a genetic mutation, in the later cases they were shown to be due to genotyping errors. The difference was observed in 5 varieties for the SNP1119_176 (Table 2). In all cases a mistaken homozygous genotype (CC) was assigned to plants studied in the stability analysis, while the correct one was heterozygote (AC). These SNP genotyping mistakes are more frequent when most samples in the plate have the same genotype, since reference genotype clouds corresponding to the three possible genotypes per SNP locus are more difficult to establish. In fact, when some of these wrongly genotyped samples were re-analyzed with samples from other plates, they were assigned the correct heterozygous (AC) genotype.

A non-redundant genotype sample was built to evaluate genetic parameters related to the discrimination power of the SNP set for grapevine cultivars. Of 200 accessions studied, 49 genotypes, corresponding to synonym cultivars, sports and wild plants, were discarded. In the resulting sample containing 151 non-redundant cultivars (Additional file 1, Table S1), allelic frequencies and several genetic parameters were determined. The MAF is a measure of the discriminating ability of the markers. In the case of bi-allelic markers, the closer MAF is to 0.5, the better. In the study, 19 SNP showed a MAF between 0.4 and 0.5, while only three SNP had a MAF below 0.1. The unbiased expected heterozygosity (He) was 0.404 ranging from 0.107 (SNP1399_81) to 0.501 (SNP581_114, SNP829_281 and VV10992) (Table 4). Only three SNP showed PIC values below 0.2, the remaining comprised between 0.2 and 0.4. These values indicate that the whole SNP set has a very high discriminating capacity for grapevine varieties, and is supported by the very low global probability of identity (PI): 1.4·10^-17^. This value is much smaller than that obtained with the 6 SSR markers approved as descriptors by the International Organisation of Vine and Wine (OIV) in the analysis of 57 unique Spanish genotypes (10^-7 ^[30]) and with 9 microsatellites in the analysis of 164 European cultivars (10^-9 ^[31]), or of 991 grapevine accessions (7·10^-12^, [23]). In contrast, the PI obtained for the 48 SNP set is larger than the value obtained with 18 microsatellites in 2,739 grapevine accessions (10^-22^, [21]), or with 34 microsatellites in 745 accessions (10^-27 ^[32]). These representative examples show that, on the average, the probability of identity per microsatellite marker is between 0.06 and 0.16 while the average in the SNP set used here is 0.445 per marker. Therefore, 3-4 SNP loci would be needed to provide the discriminating power of one microsatellite locus in grapevine. Correspondingly, the 48 SNP set would give a similar identification power as 14-16 microsatellites.

**Table 4:** Genetic parameters estimated for SNP within the 48 SNP set

	Min	Max	Average
**He**	SNP1399_81	SNP829_281	
	0.107	0.501	0.404
	
**Ho**	SNP425_205	SNP581_114	
	0.060	0.765	0.397
	
**PIC**	SNP1399_81	SNP829_281	
	0.101	0.375	0.315
	
**PI**	SNP829_281	SNP1399_81	
	0.375	0.804	0.457

He: Expected heterozygosity; Ho: Observed heterozygosity; PIC: Polymorphism information content; PI: Probability of identity.

**Figure 2:**
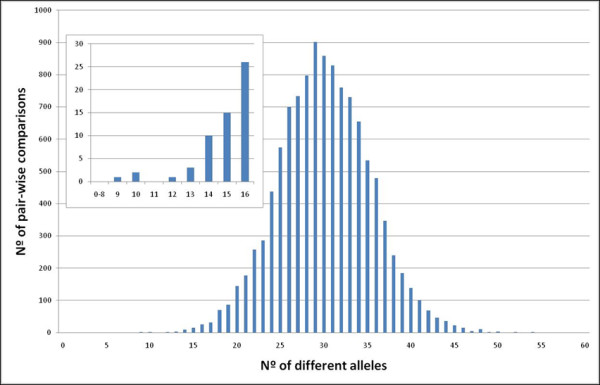
**Representation of the genetic distances among varieties**. The distances are measured in number of different alleles for the 11,325 pair-wise comparisons among the 151 non-redundant genotypes with 48 SNP. The small window is a zoom of the smallest distance zone.

The task of cultivar characterization is often related to legal issues. Of utmost importance is that in the technical test any variety has to overcome the authorization to be cultivated in many countries and that distinctness is the most important issue to be established in such tests: a variety is considered distinct if it can be clearly distinguished from all the varieties of common knowledge (Act of the International Union for the Protection of New Varieties of Plants (UPOV) Convention, 1991; http://www.upov.org/en/publications/conventions/1991/act1991.htm). The key concept for establishing distinctness is the minimum distance between varieties, which is currently established on a species by species basis, using morphological descriptors. In recent years, some efforts have been directed to incorporate molecular markers [23]. In the present study, the minimum distance among the varieties with non-redundant genotypes was determined through their pair-wise comparison and measured by the number of different alleles (Figure 2). The average difference between analyzed cultivars was 30 alleles from a total of 96 while the most different samples differed in 54 alleles. The closest cultivars found were 'Jaén Negra' and 'Zalema', which differed in 9 alleles out of the 90 that could be compared between them. These two cultivars have genotypes that are compatible with being parent/offspring, both based on microsatellites [33] as well as the SNP markers used in this study. The next closest cultivars found were 'Ciruela Roja' and 'Colgar Roja' that differed in 10 out of the 96 alleles studied. These two cultivars have recently been described as siblings of the same cross: 'Ohanes' × 'Ragol' [34]. The same occurs with 'Chardonnay' and 'Melon', which matched for 86 alleles and have microsatellite genotypes consistent with being the progeny of a single pair of parents, 'Pinot' and 'Gouais blanc' [35]. Hence cultivars studied even those genetically close, present large measured differences in the number of diverse alleles.

From the data, a very clear border exists between the highest intra-varietal variability (including here the sports) with 1 different allele and the lowest inter-varietal distance of 9 different alleles. Thus, there should not be any difficulty in establishing a minimum distance between 2 and 9 alleles for the 48 SNP set and it is large enough as to be considered conclusive for establishing distinctness in grapevine cultivars (excluding that of sports). Still a more extensive diversity study would be needed to find a more reliable minimum distance, since it could be shorter in full siblings derived from closely related progenitors as those used in current table grape breeding.

The Mendelian genetic inheritance of these 48 SNP markers has been confirmed in several previously described mapping populations. This feature also permits the genetic examination of pedigrees and parent/offspring relationships. Using the selected 48 SNP set, the total exclusion probability of paternity found for the set of 151 cultivars was high (0.9997) but the number of markers is far too small for a reliable pedigree analysis. Logarithm of odds (LOD) scores obtained for several trios ranged from 17 to 23, which are not large enough to reach final conclusions.

## Conclusions

A set of 48 single nucleotide polymorphisms (SNP) have been selected well distributed throughout the grapevine genome and tested for genetic identification purposes. The selected markers have proven to be highly stable and repeatable and also have a high discriminating power for grapevine cultivars. SNP data do not require any allele binning and allows for direct databasing and direct comparison of data arising from different laboratories. All these characteristics make our set of markers very suitable for the building of a worldwide publicly available genotype database for grapevine cultivars.

## Methods

### Plant Material and DNA Extraction

Three different cultivar sample sets and four segregating populations were used in this study. For the determination of genetic parameters concerning the 332 SNP markers under study a sample of 300 accessions including 91 wild accessions as well as wine- and table- grape cultivars (Additional file 1, Table S1) was used. These accessions are mostly maintained at the germplasm collection of "Finca El Encín" (IMIDRA, Alcalá de Henares, Madrid, Spain).

Determination of chromosomal positions of SNP markers was carried out both genetically and physically. For genetic determination four different segregating populations developed and maintained at the IMIDA (Murcia, Spain) were used: Dominga × Autumn Seedless [36], Monastrell × Cabernet Sauvignon, Ruby Seedless × Moscatuel and Muscat Hamburg × Sugraone. These mapping populations included 82, 85, 71 and 75 individuals, respectively.

The stability analysis for the selected 48 SNP set for genetic identification was conducted using fifteen cultivars, representing a high amount of variation in the cultivated *Vitis vinifera *species. Leaf material from a total of 1277 plants belonging to those cultivars was collected in 154 different plots in 7 different countries (Additional file 1, Table S2).

Analysis of genetic diversity for the selected 48 SNP set in terms of genetic identification was carried out on 200 accessions most of which came from the collection of grape varieties of the IMIDRA at 'El Encín' and the others from the CSIRO collection (Glen Osmond, Australia) (Additional file 1, Table S1).

Total DNA was extracted from frozen young leaves of each sample according to Lijavetzky et al. [37] and stored at -20°C.

### SNP Identification and Initial Genotyping

SNP discovery was approached as described by Lijaveztky et al. [13]. SNP genotyping was carried out at the Centro Nacional de Genotipado http://www.cegen.org using the SNPlex™ technology (Applied Biosystems [38]). Usefulness of the 332 SNP was studied using seven 48 SNP sets on the 300 accessions sample set. After this initial genotyping, SNP markers with a low genotyping success rate and monomorphic SNP were discarded, while the remaining ones were classified according to their minor allele frequencies.

### Determination of SNP Positions

SNP genomic locations were determined based on both genetic and physical information. Genetic positions were established using four mapping populations following a two-stage strategy. First, SNP markers were positioned on the consensus framework map developed for each cross using microsatellite markers. Molecular marker and linkage analyses were carried out according to Cabezas et al. 2006 [36] using a two way pseudo-testcross strategy [39], and the Joinmap 3.0 software [20]. In this circumstance, SNP markers can only be mapped in segregating progenies in which they segregate as aaxab, abxaa or abxab. Second, an integrated map for all progenies was built chromosome by chromosome using microsatellites as anchor markers and including all SNP segregating in at least one progeny. The integrated map was constructed using the "combine groups for map integration" function of Joinmap 3.0 [20]. Values of 3.5 for recombination frequency and 3 for LOD were used as initial mapping thresholds. For chromosomes with regions showing a low number of markers in common between the different linkage maps values were moved up to 5.0 and down to 0, respectively, allowing for map integration. For SNP showing important discrepancies in their position in the linkage maps of the different progenies physical mapping information and the "fixed order" function [20] was used to establish marker order. SNP whose inclusion led to large distortions in marker order were discarded. Chromosome names were assigned following the IGGP (International Grapevine Genome Program, http://www.vitaceae.org/index.php/ recommendations.

Physical positions of SNP markers were determined by Blat searching for their adjacent sequences on the 12× grapevine genomic sequence of the near homozygous Pinot line PN40024 [40] and http://www.genoscope.cns.fr/externe/GenomeBrowser/Vitis/. Location of markers involved in important discrepancies between genetic and physical positions was also checked on the Pinot noir genomic sequence http://genomics.research.iasma.it/gb2/gbrowse/grape/[41].

### Selection and Evaluation of a 48 SNP Set for Genetic Identification

Over 48 SNP markers were selected from the previously developed 332 according to their genotyping success rate, MAF as well as their genetic and physical positions. The last step of selection of the set for genetic identification was based on the technical requirements needed for the design of a plex for the SNPlex™ platform.

Experimental design of the stability test for the selected 48 SNP set included the analysis of 85 plants from 10 different plots (on the average) for each of 15 varieties. Plots had been planted in different years and locations in 7 different countries (Additional file 1, Table S2). Because grapevine varieties are clones, if markers used are stable, one expects to obtain the same alleles for each SNP in every plant analyzed for the same variety independently of their origin, age and location.

The discriminating power of the selected 48 SNP set for grapevine cultivar identification was evaluated with a 200 accessions sample.

Genotyping and genetic parameters were estimated from these tests. For each SNP the rate of genotyping success was calculated after excluding DNA samples that failed in the amplification of all SNP. Genotyping error was calculated based on the results obtained in different analyses: by genotyping different DNA extractions of the same plant; by genotyping different plants belonging to the same cultivar; or by studying known sports of a given genotype such as those of the Pinot family. Genetic parameters were estimated on non-redundant genotypes. Minor allele frequency (MAF), observed heterozygosity (H_o_), expected heterozygosity (H_e_) and probability of identity (PI) were calculated using the IDENTITY 1.0 tool [42] and the Excel Microsatellite Toolkit [43]. Pedigree relationships were analysed with the Cervus 3.0 software [44]. LOD scores were obtained taking the natural log (log to base e) of the overall likelihood ratios for the father-mother-offspring trios, as implemented in Cervus 3.0. [42].

## List of abbreviations used

cM: centimorgan; H_o_: Observed heterozygosity; H_e_: Expected heterozygosity; IGGP: International Grapevine Genome Program; INDEL: Insertion-deletion; LOD: Logarithm of odds; MAF: Minor allele frequency; Mb: Megabase; NCBI: National Center for Biotechnology Information; OIV: International Organisation of Vine and Wine; PI: Probability of identity; PIC: Polymorphism information content; SNP: Single nucleotide polymorphism; SSR: Simple sequence repeat; UPOV: International Union for the Protection of New Varieties of Plants.

## Authors' contributions

JAC carried out the physical and genetic mapping of the SNP, participated in the SNP selection and drafted part of the manuscript. JI carried out the stability and genetic diversity analyses and drafted part of the manuscript. DL played a part in the selection of SNP selection and characterization. MDV participated in the stability analyses. GB, VR, IC and LRG contributed in the genotyping of cultivars and progenies. AMJ participated in the SNP selection. JC generated and maintained most of the progenies. MRT assisted in the SNP selection and helped draft the manuscript. JMZ conceived the study, partook in its design and coordination and helped draft the manuscript. All authors read and approved the final manuscript.

## Supplementary Material

Additional file 1 **Supplementary Tables S1 to S5**. Table S1: Plant samples analyzed. Table S2: Plant samples used for the stability studies of the 48 SNP set. Table S3: Basic information on the 238 SNP analyzed. Table S4: Genetic maps features. Table S5: Number of progenies with heterozygous markers in at least one progenitor. Click here for file
